# Gene-level quantitative trait mapping in *Caenorhabditis elegans*

**DOI:** 10.1093/g3journal/jkaa061

**Published:** 2021-01-23

**Authors:** Luke M Noble, Matthew V Rockman, Henrique Teotónio

**Affiliations:** Institut de Biologie, École Normale Supérieure, CNRS 8197, Inserm U1024, PSL Research University, F-75005 Paris, France; Department of Biology, Center for Genomics and Systems Biology, New York University, New York, NY 10003, USA; Department of Biology, Center for Genomics and Systems Biology, New York University, New York, NY 10003, USA; Institut de Biologie, École Normale Supérieure, CNRS 8197, Inserm U1024, PSL Research University, F-75005 Paris, France

**Keywords:** genetic architecture, experimental evolution, quantitative trait, complex trait, QTL, MPP, Multiparental Populations, Multiparent Advanced Generation Inter-Cross (MAGIC)

## Abstract

The *Caenorhabditis elegans* multiparental experimental evolution (CeMEE) panel is a collection of genome-sequenced, cryopreserved recombinant inbred lines useful for mapping the evolution and genetic basis of quantitative traits. We have expanded the resource with new lines and new populations, and here report the genotype and haplotype composition of CeMEE version 2, including a large set of putative *de novo* mutations, and updated additive and epistatic mapping simulations. Additive quantitative trait loci explaining 4% of trait variance are detected with >80% power, and the median detection interval approaches single-gene resolution on the highly recombinant chromosome arms. Although CeMEE populations are derived from a long-term evolution experiment, genetic structure is dominated by variation present in the ancestral population.

## Introduction

Evolutionary biologists and complex-trait geneticists share the goal of dissecting heritable phenotypic variation down to the level of causal molecular genes and variants (Lynch and Walsh 1998; Barton and Keightley 2002). Multiparent (MP) recombinant inbred line panels are key components of the tool-kit used by geneticists to find quantitative trait loci (QTL) and study phenotypic evolution (de Koning and McIntyre 2017; Scott *et al.* 2020). Although biparental crosses have some favorable properties for mapping genetic interactions, variation is necessarily limited. MP panels increase sampling of natural genetic variation (potentially multiple alleles at the same locus), increase QTL mapping resolution and provide a more representative genetic background (Valdar *et al.* 2006; Rockman 2008). Unlike genome-wide association studies (GWAS) in natural populations of often uncertain demographic and environmental evolutionary history, MP panels in tractable organisms serve also as models of phenotypic evolution, allowing highly replicated measurement of traits, including individual breeding values, systematic manipulation of the environment, and control for potentially confounding environmental covariates.

We have introduced the *C. elegans* multiparental experimental evolution (CeMEE) panel as the first MP panel for this model organism (Noble *et al.* 2017). Natural *C. elegans* populations are generally depauperate of DNA sequence diversity due to a history of predominant self-fertilization together with linked selection and recent local population expansions (Andersen *et al.* 2012; Rockman *et al.* 2010; Cutter *et al.* 2009). *C. elegans* holocentric chromosomes show lower DNA sequence diversity in central regions with lower meiotic recombination rates, as expected from evolutionary theory (Rockman and Kruglyak 2009), and many other genomic features such as repeat content, gene density, and gene essentiality also covary with recombination rate (Cutter 2015).

**Figure 1:**
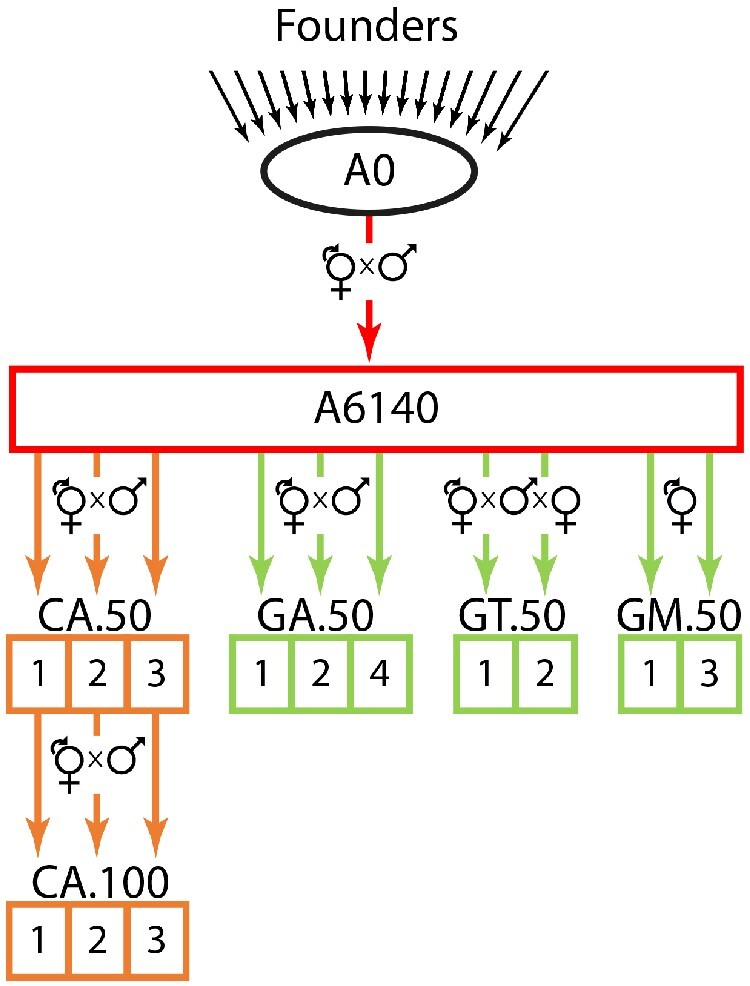
Experimental evolution scheme. Colors indicate environmental and reproductive system regimes: black for derivation of the hybrid androdiecious (hermaphrodite/male) population A0 from inbred founders, red for domestication under mixed selfing and outcrossing for 140 generations (resulting in the A6140), orange for continued evolution in the standard environment (three lineages, sampled at 50 and 100 generations), green for evolution in changing environments under androdioecious, trioecious (male/female/hermaphrodite) or monoecious (hermaphrodite) mating systems (50 generations). In each regime, samples from replicate populations (numbered in boxes) are periodically frozen for contemporaneous characterization of ancestral and derived characters. CeMEE RILs were derived from A6140 and all descendant lineages shown.

CeMEE is unique among MP panels in that it represents a sample of genotypes from large experimental evolution populations where the demographic and environmental history in the lab is known, and where the relationship between traits and fitness can be assayed contemporaneously in ancestral and derived populations. As a consequence, natural selection and genetic drift should be amenable to explicit modeling in the context of evolving genetic architectures (Teotónio *et al.* 2017). Comparison of outbred experimental evolution populations and the inbred lines derived from them has, for example, allowed us to determine that the evolution of the multivariate genetic variance-covariance structure of locomotion behavior is compatible with phenotypic stasis (Mallard *et al.* 2019), and to study the population genetics of adaptation to changing environments (Guzella *et al.* 2018). CeMEE is derived from an intercross of 16 founders, a 140 generation lab domestication phase, and then 50 to 100 generations of subsequent experimental evolution under variable sex ratios and breeding mode (self-fertilization and outcrossing), at census population sizes of 10^4^ and effective population sizes of around 10^3^, in a defined, stable, and unstructured laboratory environment (Figure 1).

Here, we report a 50% expansion of the CeMEE panel, and we investigate properties of the new panel for discovery of additive and interactive QTL. We selected 763 genome-sequenced RILs for inclusion in CeMEE v2, from more than 1000 RILs in our cryopreserved collection, bringing improvements in power to map and localize QTL. Simulations show that power for additive QTL is >80% for loci explaining 4% of trait phenotypic variance, loci that can often be mapped at gene-level resolution in regions of high recombination. These are values comparable to those of the Drosophila Synthethic Population Resource (DSPR), a powerful metazoan MP resource (King *et al.* 2012a, 2012b). In the previous CeMEE iteration, we showed that the genetic architectures for fitness (fertility) and adult hermaphrodite body size are characterized by both high additive and epistatic polygenicity, and strong oligogenic sign epistasis (Noble *et al.* 2017). Here we show, using simulations, that statistical power to detect epistasis is limited to strong pairwise additive interactions (>80% for interactions explaining at least 7% of trait variance).

The expanded CeMEE also gives us access to questions about the evolution of population-genetic structure, selective sweeps, and new mutation. Although experimental evolution imposes clear population structure across the CeMEE as a whole, the extent of differentiation is relatively weak considering the now more than 500 generations that in sum separate the replicated populations from which RILs have been derived; this stands in contrast with the substantial population structure found in nature. At the same time, our data reveal several regions of the genome that have experienced rapid changes in haplotype frequencies, consistent with positive selection acting on standing variation during experimental evolution. Finally, new data and expansion to new replicate populations allowed us to gain insights as to the rate, molecular spectrum and frequency dynamics of *de novo* mutation appearing during lab evolution. Our findings show some similarity to those found in mutation accumulation experiments, where selection is minimized (Saxena *et al.* 2019).

## Materials and methods

### Experimental evolution and recombinant inbred lines

The CeMEE panel comprises recombinant inbred lines sampled from multiple populations, derived from a common ancestor and evolved under a discrete life-cycle (Figure 1). Building on a previous release (Noble *et al.* 2017), we sequenced (or resequenced to greater depth) an additional 455 RILs. Of these, 169 lines come from three new populations (control androdioecious CA[1-3]100, where [1-3] designates the population replicate), evolved for a further 50 generations from the CA[1-3]50 lineages under our standard experimental evolution conditions. Other RILs come from populations already reported: A6140, a lab domesticated population derived from the A0 hybrid population (itself derived by parallel intercrosses among the 16 founders) by 140 generations of experimental evolution (Teotónio *et al.* 2012); and GA[1,2,4], GT[1,2] and GM[1,3], androdioecious, trioecious and monoecious populations, respectively, evolved in gradually increasing NaCl concentrations (Theologidis *et al.* 2014). In brief, our standard laboratory environment is characterized by a constant census size, achieved by seeding each of 10 plates per population with 1000 active, synchronized L1 larvae, growth at 20 C in the presence of excess *Escherichia coli*, and discrete generations enforced by bleaching of reproductively mature adults at 4 days post seeding (Teotónio *et al.* 2012). Plates were filled with 28 mL of Nematode Growth Medium lite (NGM-lite, US Biological) where NaCl concentration was 25 mM (for A6140 and all CA populations), or supplemented with NaCl reaching a maximum concentration of 305 mM for the GA, GT and GM populations from generation 35 to generation 50 (Theologidis *et al.* 2014). As before, the new RILs from CA[1-3] were derived by sampling single hermaphrodites from populations and selfing for 10 generations before preparation of genomic DNA and cryopreservation. The number of RILs derived (sequenced, post-quality control, or currently in cryopreservation but unsequenced) by population is shown in Table 1.

**Table 1:** Numbers of recombinant inbred lines derived from experimentally evolved populations in CeMEE v1 (Noble *et al.* 2017) and v2 (-IF; the total number of sequenced lines before application of an Inclusion Filter based on quality control and relatedness), and the total number of replicated, cryopreserved RILs (CeMEE v2, plus additional lines with no sequencing data at present)

Source population	v1	v2 (–IF)	cryo.
A6140	178	251 (263)	303
CA[1–3]50	118	152 (155)	152
CA[1–3]100	0	144 (168)	318
GA[1,2,4]50	127	133 (163)	154
GT[1,2]50	79	78 (88)	78
GM[1,3]50	5	5 (100)	5
	507	763 (957)	1010

Populations derived from A6140 are formatted as TMRG, where *T* is evolution treatment (here, Control conditions or Gradual adaptation to a moving optimum), *M* is mating system (Androdioecious, Trioecious, or Monoecious), *R* is replicate number, and *G* is the number of generations from A6140.

### Genome sequencing and variant calling

RIL genomic DNA was prepared using the Qiagen Blood and Tissue kit. Library preparation and sequencing was carried out at the Beijing Genome Institute on the Illumina HiSeq X Ten or BGIseq platforms with, respectively, 150 and 100 bp paired-end reads to a mean depth of 4.2× and 22× (estimated from per base mapped read depth at the central recombination rate domain of chromosome I).

Given the large increase in samples, sequencing data, and algorithmic improvements, we revisited calling of founder variants and potential *de novo* mutations by jointly calling all 957 RIL samples with any sequencing data with the 16 founders against the WS220 N2 reference (GATK v. 4.1.7.0; McKenna *et al.* 2010; DePristo *et al.* 2011). We defined an updated set of 386,584 stringently filtered founder diallelic single-nucleotide variants (SNVs) across all samples (QUAL > 1000, <50% heterozygous or missing calls in founders, MQ > 40, DP median absolute deviation < 0.995 percentile, SOR < 3, QD ≥ 15; Supplementary File S1).

Missing data in RILs were imputed by Hidden Markov Model (HMM), as in Noble *et al.* (2017). Following quality control (see RIL quality control and filtering below), we took the subset of 354,063 SNVs segregating in 763 RILs as our base of analysis (Supplementary File S2; coded [0,1] against the N2 reference genome. Genotype calls of less certain zygosity, i.e., intermediate HMM probabilities and GATK heterozygous calls, are coded >0 and <1, of which the per line median is 0.4%).

Heterozygous calls were elevated among CA100 RILs, mostly for population replicate CA3100 (e.g., 93% of 135 lines sequenced to >10× coverage showed both reference and alternative alleles at >20% of segregating sites). Minor allelic proportions were generally low, however (11% of these 135 lines had mean proportions >20%). We removed extreme cases as part of our quality control (see RIL quality control and filtering below).

We considered RIL variant calls absent from founders as potential new mutations, conservatively adopting the following criteria: mapping quality MQ ≥ 40, quality/depth QD ≥ 5, total depth DP ≥ 30, positive deviation from median depth quantile < 0.95, strand odds ratio SOR < 4, read position rank sum > –4, heterozygote frequency < 10%, and at least 3 homozygote samples (a frequency of just over 0.3%). This filtering biases detection to mutations that arose early in evolution and were selected, directly or indirectly; we exclude rare mutations, such as those arising during RIL construction, as they are more likely to be false positives. We also excluded all sites where the founders vary in an independent data set (*C. elegans* natural diversity resource [CeNDR] isotypes in soft-filtered release 20180527) as likely false negatives (Cook *et al.* 2017), and examined 12,826 remaining candidate new diallelic SNVs with no missing data. Of these, 7590 variants occur at sites that are invariant among all CeNDR isolates (Cook *et al.* 2017).

### RIL quality control and filtering

Quality control considered homozygosity, sequencing depth, and haplotype reconstruction likelihood, and we additionally filtered on relatedness to remove very closely related lines. From 957 sequenced lines, identity at segregating sites was thresholded to a maximum of 90% (removing all but 5 lines from monoecious GM populations, and 49 lines from other populations), minimum expected sequencing depth of at least 0.1× (*n*  =  2) and, based on segregating sites covered by at least 3 reads, a maximum of 20% where both reference and alternate alleles were seen with mean minor allele frequency > 20% (*n*  = 24). After sequence and genotype filtering, outlier lines with haplotype reconstruction posterior log likelihoods (see below) beyond the 0.1 percentile in deviation from the population median for more than three chromosomes were also excluded (*n*  = 13). Haplotype reconstruction outliers showed no significant population bias; they were consistently associated with a high minor allele proportion at heterozygous sites, though not always a high frequency of heterozygous sites genome-wide.

As a result of filtering, 35 lines in CeMEE v1 were dropped from v2 (20 replaced with closely related lines with greater higher sequencing depth, nine lost from cryogenic storage, and six haplotype reconstruction outliers).

### Haplotype reconstruction

**Figure 2:**
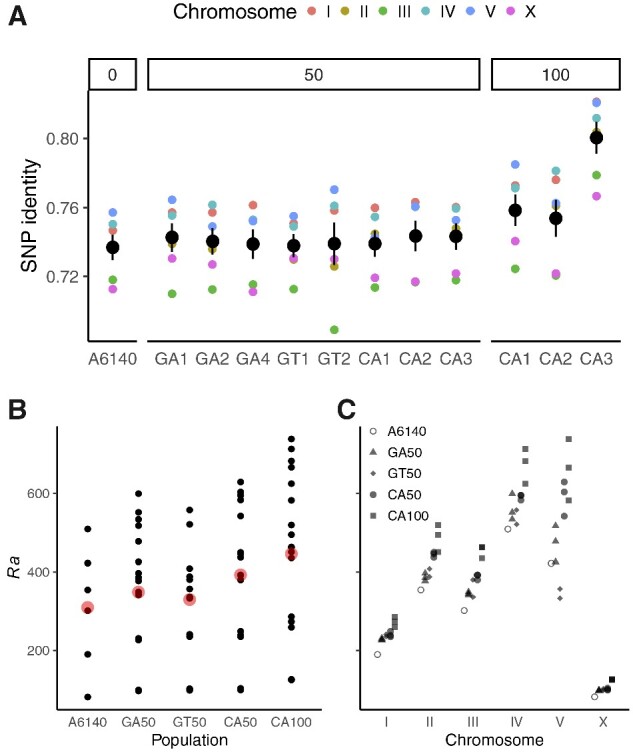
(A) Genetic relatedness within population replicates, grouped by generation from A6140 (mean and standard error of pairwise identity among lines at segregating sites for each chromosome). (B–C) Realized map expansion across experimental populations (B) and chromosomes (C). Each point is a single value for a chromosome of each replicate population, with the grand mean overplotted in red in (B). Map expansion increases with generation ($p<10^{-35}$ by Poisson linear model), and for all chromosomes, though variably so (*r*^2^ > 0.77 for chromosomes other than V (0.58) and the outlying IV (0.37), the latter of which carries a very highly recombined haplotype within the right arm piRNA cluster (Chelo and Teotónio 2013; Noble *et al.* 2017).

As in Noble *et al.* (2017), we used the RABBIT framework (Zheng *et al.* 2014, 2015) for RIL haplotype reconstruction from founder genotypes and the N2/CB4856 genetic map (with all non-homozygous genotype calls set as missing data). For each chromosome and population replicate, we estimated maximum likelihood map expansion (*Ra*) by Brent search (Brent 2002) under the fully dependent homolog model (*depModel*) for each line (assuming full homozygosity, and founder and RIL genotype error rates of 0.5%) in Mathematica 11.0.1 (Wolfram Research, Inc. 2016). Per marker haplotype posterior probabilities, per line likelihoods, and Viterbi-decoded paths (Viterbi 1967) were then calculated using this value. Outliers (as defined above) were removed and the process was repeated to arrive at final *Ra* values shown in Figure 2.

Haplotype representation and diversity in Figure 5 was summarized from Viterbi paths in 5 kb windows (excluding the 5 GM lines). Figure 5B shows entropy $S=-\sum_{i=1}^{f}\sum_{j=1}^{n}p_{ij}log_{2}p_{ij}$, with frequency *p* of haplotype *i* in population *j* summed over *n* populations and *f* founders (Atwal *et al.* 2007). Haplotype frequencies were evaluated under expectations of pure drift (*f *=* *1/16 for equal founder haplotype frequency) or selection of the unique founder haplotypes (diallelic SNV genotypes in each window).

### Population structure

Additive genetic relatedness matrices (**A**) constructed from segregating SNVs (excluding ambiguous imputations) were decomposed with the base R function *prcomp* (R Core Team 2018). Given the matrix of *n* lines by *m* SNVs, genetic similarity was calculated by scaling each marker to mean 0 and variance 1, with $A=XX^{T}/m$ scaled to a mean diagonal value of 1. Populations were decomposed jointly across chromosomes or recombination rate domains (Rockman and Kruglyak 2009), excluding the 5 GM RILs. *F_ST_* was calculated using Hudson’s estimator (Bhatia *et al.* 2013).

### QTL mapping simulations

#### Additive tests

Additive QTL were simulated varying the variance explained by a single focal marker (0.01–0.12) and a background polygenic component (spread over 50, 100, or 500 markers) with total heritability set to 0.5 and effect sizes drawn from the standard normal distribution. We fit single marker linear mixed-effects models (R package GridLMM; Runcie and Crawford 2019) to test for fixed SNV effects. With *y* the mean-centered vector of simulated phenotypes for *n* individuals:

(1) $$\begin{array}{cc} \;y & =X\beta +Zu+e\\ & u∼N(0,\sigma_{g}^{2}A)\\ & e∼N(0,\sigma_{e}^{2}I) \end{array},$$

where *X* and *Z* are design matrices for fixed and random effects *β* and *u*, respectively. The vector of breeding values *u* is assumed to be normally distributed with mean 0, genetic variance $\sigma_{g}^{2}$, and variance-covariance *A*, generated from markers pruned of complete LD (marker set 1, $r^{2}<0.99$). Residuals are assumed to be normally distributed with mean 0, variance $\sigma_{e}^{2}$, and uncorrelated (*I* is an *n *×* n* identity matrix). For each heritability scenario, defined by focal marker variance explained (*h*^2^ or, equivalently, *r*^2^) and the number of markers in the polygenic component, we ran 1000 simulations sampling loci with minor allele frequency (MAF) > 5% from the LD pruned genotypes. Significance was set at α=10% based on the effective number of markers (*M_eff_*) given observed linkage disequilibrium (LD). We used MultiTrans to estimate *M_eff_*, which varies with LD, genetic relatedness and trait heritability (Joo *et al.* 2016). Averaging across runs for 12 heritability scenarios (200,000 samples, 1000 SNV windows; fixing background polygenicity at 100 markers, total $h^{2}=0.5$ as above) gave a threshold of $p<3.23\times 10^{-6}$. Detection intervals were defined by markers with LOD (logarithm of odds) score within 1.5 units of the peak QTL marker, or flanking markers at minimum. Local haplotype structure can lead to a nonmonotonic relationship between physical distance and association statistics. To better account for this when defining QTL confidence intervals we ranked all markers by LD with a peak marker in 300 kb windows and calculated boundaries using this ranking. A single QTL at most was defined per window, and contiguous intervals were then merged across windows offset by half. Mean interval size was insensitive to the window parameter over reasonable parameter ranges given the observed LD decay (e.g., 200–500 kb) shown in Noble *et al.* (2017). This procedure yielded conservative confidence intervals, with simulated variants falling within the peak interval more than 98% of the time when detected. Figures show summary statistics for binned realized marker *h*^2^ (i.e., linear regression adjusted *r*^2^ for the focal marker), which differs slightly from simulated *h*^2^ due to sampling, yielding at least 800 simulations for each heritability scenario. Additive QTL simulation code is in Supplementary File S3.

#### Interaction tests

The power to map interactions was tested by sampling marker pairs and simulating phenotypes with a given epistatic heritability—the proportion of variance explained by interaction of the focal markers—with effects drawn from the standard normal distribution (i.e., polygenic variance was not simulated, and main effects were unconstrained). Markers were pruned of strong local LD ($r^{2}<0.5$, MAF > 5%), and sampled pairs were tested only if all four genotype classes were present in at least 3 lines. With mean-centered phenotype vector *y* and marker genotypes *i* and *j*, a full model *M_1_* was tested against the null additive model *M_0_* by likelihood-ratio (LR) test:

(2) $$\begin{array}{cc} M_{0}: & \;y=X_{i}\beta_{i}+X_{j}\beta_{j}+e\\ M_{1}: & \;y=X_{i}\beta_{i}+X_{j}\beta_{j}+X_{ij}\beta_{ij}+e \end{array}.$$

Empirical *P*-values were obtained by bootstrap, controlling for main effects (unlike permutations of trait values, which simultaneously vary main and interaction effects) (Bůžková *et al.* 2011). Responses were sampled from the observed *M_0_*, and LRs comparing *M_1_* and *M_0_* fit to the resampled data were then stored. For each heritability level, at least 10,000 simulations were run, taking 100 null LRs at each test. Test statistics are $\chi^{2}$ distributed with a mixture of 0 and >0 degrees of freedom (Self and Liang 1987). This mixture was estimated from pooled null statistics, stratified by joint MAF decile, for conversion of alternative LRs to *P*-values (Listgarten *et al.* 2013; Casale *et al.* 2015). Code to simulate epistatic QTL and generate null samples is in Supplementary File S4, code to generate *P*-values is in Supplementary File S5.

Genome-wide significance was declared at α=10% after Bonferroni correction for multiple tests based on the effective number of markers. We use a conservative Bonferroni correction over other multiple testing adjustments, as interaction detection is known to be more prone to false positives (Wei *et al.* 2010, 2014). *M_eff_* was estimated directly from markers (i.e., not controlling for additive genetic relatedness, as was done for the additive simulations), defined here for this smaller set as the number of eigenvalues of the marker correlation matrix *R* that explain at least 99% of the variance. Covariance matrices sampled from an unobserved population are typically biased in the distribution of eigenvalues when the number of variables (markers) *p* is much larger than the number of observations *n* (Meyer 2016). Following Davis *et al.* (2016), we applied the Ledoit-Wolf shrinkage estimator to *R* before eigenvalue decomposition, yielding *M_eff_* = 6236 (of 6428 markers) and a conservative adjusted significance threshold (since not all marker pairs were tested) of $0.1/((M_{\mathrm{eff}}(M_{\mathrm{eff}}-1))/2)=5.14\times 10^{-9}$. The figures show summary statistics for binned realized interaction *h*^2^ (linear regression adjusted *r*^2^).

### Data availability

All raw read data are available from the NCBI SRA under BioProject PRJNA557613. Sequencing and other metadata are available from lukemn.github.io/cemee. Supplemental files and code are available at FigShare. They are also available from the CeMEE github, with genotypes in WS220 and WS245 reference genome coordinates, and input files for the R packages *qtl* and *qtl*2.

Supplementary Files S1 and S2 contain processed genotypes, Supplementary File S3 contains R (R Core Team 2018) code for additive QTL simulations, Supplementary Files S4 and S5 contain Python 3 (Python Software Foundation) code for running epistatic QTL simulations and generating empirical *P*-values, Supplementary File S6 contains marker positions in genetic distance, Supplementary File S7 contains an additive genetic relationship matrix, Supplementary File S8 contains recombination rate domain boundaries. See the Data Document for more details. All lines (founders, CeMEE v2 RILs, and additional RILs not yet sequenced) are cryopreserved in replicated 96-vial plates and are freely available for noncommercial purposes.

Supplementary material is available at figshare: https://doi.org/10.25387/g3.12293138.

## Results and discussion

### Panel composition

Through derivation of inbred lines from new experimentally evolved populations and (re)sequencing of RILs from existing populations, we expanded the CeMEE panel by more than 50% over the version 1 release (Noble *et al.* 2017). From 957 sequenced lines, we retained 763 after application of an inclusion filter based on genomic SNV relatedness and quality control on sequencing depth, zygosity (whether due to residual heterozygosity, or line or DNA contamination during inbreeding and sequencing), and haplotype reconstruction likelihood (Table 1).

### Improved QTL mapping power and resolution

The aims of a long-term evolution experiment and those of QTL mapping are not fully aligned. Drift and selection acting on standing genetic variation will lead to differentiation of populations, and potentially to loss of genetic diversity until mutation-selection-drift equilibrium. We showed previously extensive divergence from the founders during the initial MP funnel and domestication phases leading to A6140, with more than 32,000 SNVs fixing. Few large-scale hard sweeps were observed, however, with the loss of founder singletons accounting for around 80% of these cases, and over 97% of the autosomal genome remaining genetically variable (at 20 kb scale). Although the new CA100 populations are more homogeneous than their CA50 progenitors (Figure 2A), suggesting reduced *Ne*, we continue to see 0 fixed SNV differences between any pair of replicate populations derived from A6140, consistent with an absence of bottlenecks and a highly polygenic architecture for fitness.

Mapping resolution is limited by effective recombination. With the addition of 144 CA[1-3]100 RILs alone from a further 50 generations of evolution (sampling some 750,000 crossover events per autosome in outcrossing populations), we expect gains in resolution, subject to the maintenance of recombinant diversity within and among population replicates. Potential gain (or loss) of power due to atomization of linked quantitative trait nucleotides of antagonistic (or similar) effect is an empirical question (Bernstein *et al.* 2018). Realized genetic map expansion (*Ra*) estimated during joint haplotype reconstruction shows continued gains in recombination in the CA[1-3]100 (Figure 2B). These are seen for all chromosomes (Figure 2C), and are of a similar magnitude to the preceding 50 generations of adaptation from A6140 to the CA[1-3]50 populations. The per generation and chromosome increase in *Ra* is 1.21 for CA50s versus 1.15 for CA100s, with progressive underestimation of the true map expansion expected over time (Noble *et al.* 2017).

**Figure 3:**
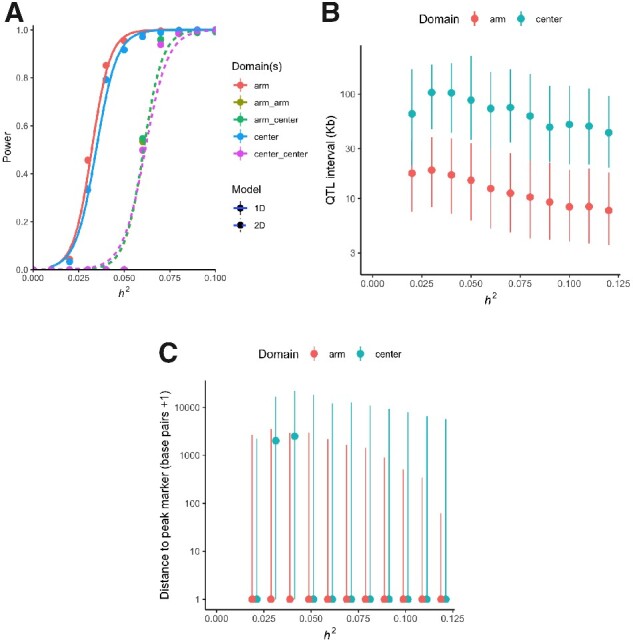
(A) Detection power for additive (1D; single marker linear mixed-effects model) or interaction effects (2D; linear regression for a single marker pair), at 10% FDR based on the effective number of tests. Results (mean and standard error) are split by chromosome recombination rate domain(s) of the simulated site(s). (B) QTL detection intervals (median and interquartile range of markers within 1.5 LOD units of the peak marker) for the single-marker additive test. (C) Median and interquartile range for the distance of the true simulated site from the peak QTL marker. The *x*-axis shows simulated marker *h*^2^ (equivalently, *r*^2^).

Simulations of additive QTL show 80% power for SNVs explaining around 4% of the phenotypic variance (Figure 3A). Mapping resolution on the recombination- and variant-rich arms approaches single genes for QTL of large-effect: for SNVs explaining 3–10% of trait variance, median 95% (LOD 1.5 drop) confidence intervals are 8.3–18.7 kb (Figure 3B, and the median distance of the QTL peak from the true QTN is 0 bp over the same heritability range (Figure 3C). Long haplotypes on the chromosome centers limit resolution, and certainty, with median detection intervals of 48-103 kb and peak distance to the true QTN 0–2.5 kb over the same heritability range. In general, the minor allele frequency (MAF) spectrum is relatively flat beyond 5%, and in this range the effects of MAF on mapping power are limited (Supplementary Figures S1 and S2).

Detecting epistasis at the QTL level is difficult due to scaling of the number of tests and joint allele frequencies (Phillips 2008). Only cases of second-order epistasis with strong associations, such as those mapped previously for hermaphrodite fertility and body size in CeMEE v1 (Noble *et al.* 2017), are likely to be reliably detected (>80% power for interactions explaining 7% of trait variance).

### Population structure

The structured nature of the panel presents some challenges for mapping the causal basis of trait variance, particularly for redundant genetic architectures of highly polygenic traits drifting within replicate experimental lineages. For less polygenic traits, the influence of phenotype and genotype confounding on false positives weakens as the number of discrete populations increases (Rosenberg and Nordborg 2006). Structure due to experimental evolution is, of course, known by design, and in the simplest case can be handled by conditioning on population means (on the assumption of consistent directional effects across populations). Subtler patterns of realized genetic relatedness, approximated by genome-wide SNV data, can be accounted for in the standard linear mixed-effects model framework.

**Figure 4:**
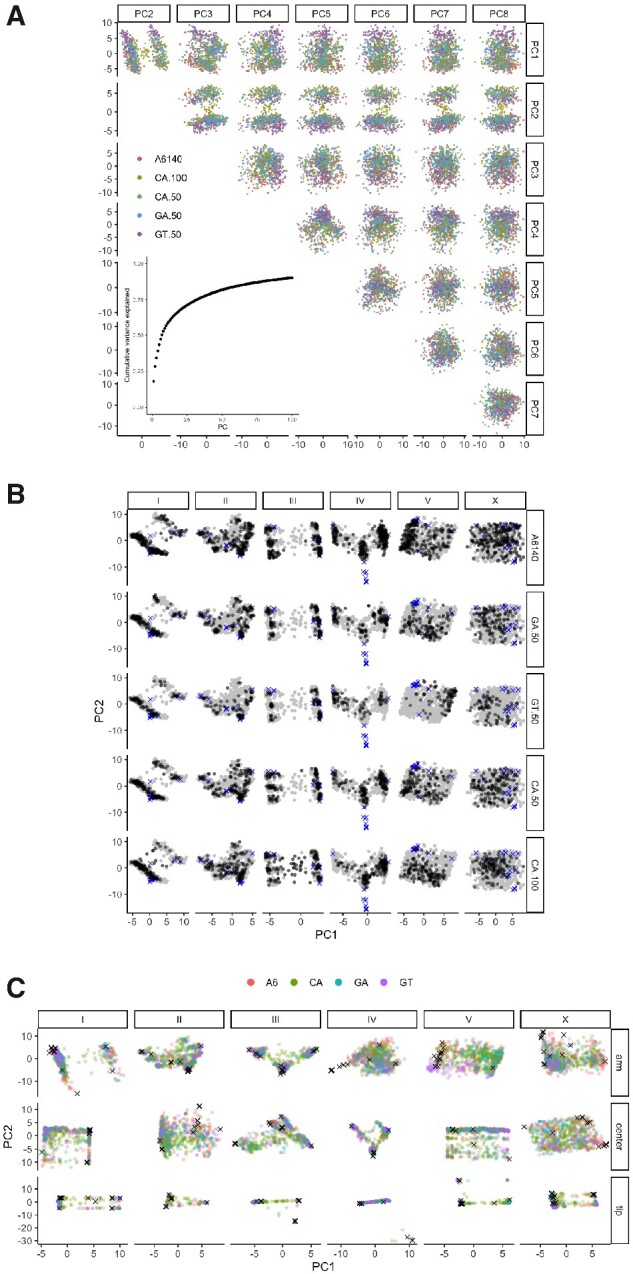
Genetic structure is not dominated by experimental structure. (A) The first eight principal components (PCs) of the additive genetic relatedness matrix (accounting for almost half of the variance), colored by population. The inset shows the cumulative proportion of variance explained by the first 100 PCs. (B) Populations show relatively consistent structure, with the exception of chromosome V for GT populations due to introgression of a sex-determining allele. The top PCs for each chromosome are shown by experimental population (replicates combined). The full space occupied by populations (gray background points) and founders (× symbols) is shown across all panels. Variance explained for the populations ranges from 17% for chromosome X to 57% for IV. (C) As in B, with populations overplotted (CA50 and CA100 pooled), and stratified by recombination rate domain. All values are multiplied by 100.

We previously showed that while experimental design has, as expected, generated significant structure (Chelo and Teotónio 2013; Noble *et al.* 2017), this is not reflected in the major axes of variation (with the exception of GT populations, which show strong differentiation for an introgressed sex-determining allele on chromosome V captured by the first principal component, see Supplementary Figure S4A). Revisiting this with new data, we again see that the principal components of additive genomic relatedness show strong structure that varies within and across chromosomes, but is largely consistent across populations (Figure 4, and see multidimensional scaling in Supplementary Figure S3).

**Figure 5:**
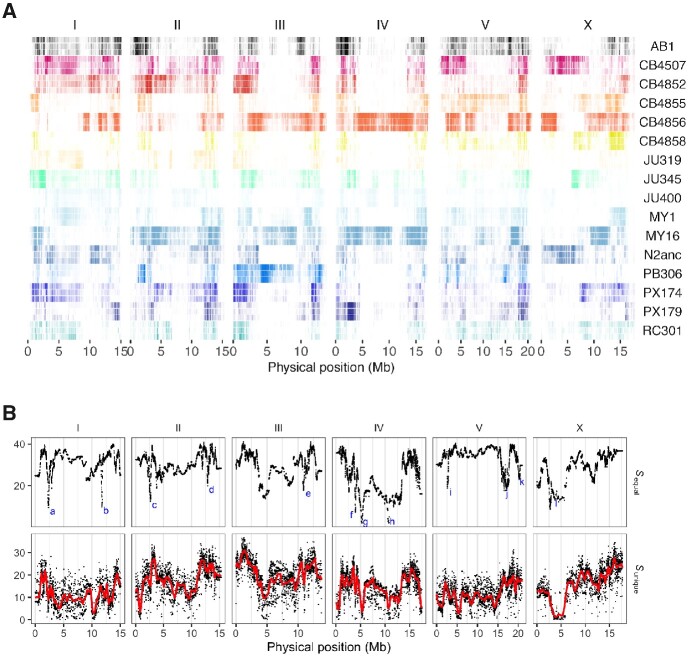
(A) Founder representation across CeMEE populations. For each of the 16 founders, the composition of reconstructed haplotypes is shown for A6140 (top), CA[1-3]50 (middle) and CA[1-3]100 (bottom) RILs, averaging over populations for CA lines. Identity at segregating markers in 5 kb windows is plotted, color intensity scales with frequency (see Supplementary Figure S5A for quantities). (B) Upper row: haplotype divergence across all CeMEE RILs in each 5 kb window (entropy, high values indicating low divergence), against the expectation of equal representation of all 16 founders. Outliers highlighted span known loci *zeel-1*/*peel-1* (a), *fog-2* (k; an experimental artifact from introgression of a null allele into GT populations), and *npr-1* (l). Other labeled, well-localized peaks of divergence correspond to selection of (b) CB4856 haplotypes upstream of *vab-10*, (c) hyperdivergent CB4852 haplotypes, (d) hyperdivergent MY16 haplotypes, centered on *gsy-1*, (e) MY1 haplotypes spanning *vab-7*, (f-h) long, recombined MY16 and CB4856 haplotypes nearing fixation, (i) hyperdivergent CB4856 haplotypes, and (j) MY16 haplotypes with unique variants in *srr-3*, *cpr-2* and four other genes. Second row: as above, but against the expectation of equal proportions of the unique SNV founder haplotypes observed in each window, with a locally-weighted (LOESS) polynomial regression.

Haplotype divergence varies markedly across and within chromosomes (Figure 5). This is due in part to recombination rate variation within chromosomes, existing population genetic structure in the founders (Andersen *et al.* 2012), and selection during experimental evolution. We noted fixation of N2-like haplotypes across a large region of chromosome X centered on *npr-1* (4.77 Mb; de Bono and Bargmann 1998; Andersen *et al.* 2014; Sterken *et al.* 2015) and near-fixation of a single founder haplotype around the *zeel-1/peel-1* selfish genetic element on chromosome I (the N2-type JU345 haplotype, around 2.34 Mb; Seidel *et al.* 2008), suggesting compatibility state is not the sole factor determining evolution of this locus. We also note similar levels of divergence, due to strong selection of single-founder haplotypes, at several uncharacterized loci across the autosomes (Figure 5B).

### New mutations

**Figure 6:**
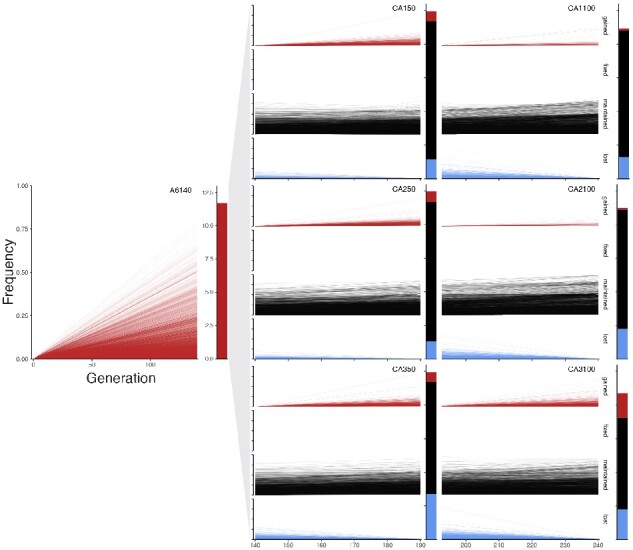
Frequencies of candidate new mutations estimated from RILs of A6140 and three derived control (CA) replicate lineages sampled at G + 50 and G + 100. Of 12,826 filtered diallelic SNVs, the majority (11,695) arose sometime before sampling in A6140, and many were maintained in CA populations (10,511 detected in at least one lineage at generation 50 and 100, 5293 detected in all six). For each derived population, variants are split and colored by class (gained, fixed, maintained or lost, labeled at left for CA100 populations). All unlabeled axes are consistent across plots.

We examined 12,826 filtered diallelic SNVs present in CeMEE RILs, but not founders, as potential *de novo* mutations (excluding all sites that vary in the CeMEE founder CeNDR isotypes). We refer to these here for brevity as ’new mutations’ and compare them with standing genetic variation (SGV; 372,578 equivalently filtered SNVs present in founders). Most detected new mutations arose before sampling of the A6140, during the initial phase of lab adaptation Figure 6A. Losses of new mutations outweighed gains in subsequent generations, particularly under the novel NaCl environment (Supplementary Figure S6). Although the candidate mutations here are clearly a biased subset, exposed to potentially hundreds of generations of selection, as an initial analysis we examined characteristics of new mutations and SGV to see if they differ on average.

First, we found that new mutations are depleted on the X chromosome, which is expected on grounds of differential dominance (if most nonneutral mutations are deleterious and recessive) and effective population size (mutations will arise less frequently, and be lost by drift more often, on the X in the outcrossing CeMEE populations; Fisher’s exact test $p<10^{-56}$ for *X* vs. autosomes, ratio of new mutations to SGV on the *X* = 0.57, with the next lowest value at 0.77 for chromosome IV). Second, although new mutations are found at similar frequencies to SGV across recombination rate domains, on average, they are less uniformly distributed across autosomes (the Gini coefficient, a measure of inhomogeneity, is higher for all; *P *<* *0.02 from 1000 SGV subsamples at α=5%). Third, transition/transversion ratio is much lower for new mutations (0.99 vs. 1.27 for SGV; $p<=10^{-4}$ from 10,000 subsamples). Mutations that have arisen in the laboratory—from mutation accumulation experiments, or during domestication of the N2 reference strain—are known to have a lower ratio (around 0.7-0.8) than that of SGV (Saxena *et al.* 2019). Ts/Tv for new mutations falls monotonically with increasing variant quality (which is largely driven by allele frequency), while that of SGVs rises slightly (e.g., 0.88 at $\mathrm{QUAL}>10^{5}$ for new mutation, vs. 1.30 for SGV). Fourth, predicted functional effects differ: the proportion of new mutations with low predicted impact (synonymous codon changes) is more than two-fold lower than for SGV, but it is slightly greater for high predicted impact (Fisher’s exact test *P *<* *0.0025), and many more variants fall within introns (around one-third for SGV, and half for new mutations; Fisher’s exact test $p<10^{-85}$).

## Conclusions

We have expanded the CeMEE panel by more than half, with recombinant inbred lines drawn from discrete populations 50–100 generations from common ancestry. Despite the highly structured nature of the panel, allele frequency differentiation among populations is limited, and most of the dominant axes of variation stem from genetic structure already present in the founders and maintained through hundreds of generations under varying evolutionary regimes.

The majority of candidate new mutations detected here are shared across populations, having arisen in the 200 or so generations between isolate hybridization and sampling of the A6140 lab-adapted population. Although at least some of the variants, present across multiple samples after stringent filtering, are likely to have been maintained by direct or indirect selection, the number is unexpectedly high. Assuming a haploid base substitution mutation rate of $2.5\times 10^{-9}$ (Saxena *et al.* 2019) and an effective population size of 1000 (Chelo and Teotónio 2013), around 70,000 new mutations are expected to have arisen in the A6140 population in total, just six times the number detected. Further work on haplotype dynamics, linkage disequilibrium, and fitness consequences will be required to refine these estimates.

QTL mapping power and resolution will continue to improve as additional RILs are sequenced, though returns diminish. Mapping efforts for highly polygenic traits may be better served by focusing on a single recombinant source population at a single point in time, where genetic background is more consistent. QTL mapping resolution in the low recombining centers can, however, be much improved by manipulating recombination. With this goal in mind, we have introgressed an allele of the recombination modifier *rec-1*, which homogenizes recombination rates along the chromosomes without affecting total genetic map length (Zetka and Rose 1995; Chung *et al.* 2015), into the lab-adapted A6140 population.

Another venue for improvement is integration between CeMEE and the *C. elegans* natural diversity (CeNDR) panel, a growing collection of wild isolates and genotype-phenotype associations (Cook *et al.* 2017). QTL discovery in CeNDR followed by validation in CeMEE RILs is a potentially powerful approach, allowing rapid fine-mapping and interaction testing (where causal variation is present in CeMEE founders) or a useful filter on CeNDR variation to facilitate follow-up (where additive QTL do not replicate in CeMEE). Comparing QTL mapping results for the same traits from CeMEE and CeNDR also holds promise to understand the importance of dominance and epistasis in adaptive responses (Barton 2017), particularly the generation and maintenance of genetic incompatibilities, as the former suffers from inbreeding depression (Chelo *et al.* 2014, 2019), whereas the latter shows outbreeding depression (Dolgin *et al.* 2007). Ideally, in order to address questions about outbreeding and inbreeding depression, namely how they relate to DNA sequence diversity, new CeMEE panels should be derived from new, highly diverse sets of natural isolate founders (Crombie *et al.* 2019). The panel’s greatest utility for understanding trait genetics and evolution may be realized as molecular, cellular and organismal phenotypes are generated and analyzed jointly (Houle *et al.* 2010), as we have started to do elsewhere (Guzella *et al.* 2018; Mallard *et al.* 2019).

## Supplementary Material

jkaa061_Supplementary_Data
